# Inhibition of ER stress improves progressive motor deficits in a REEP1-null mouse model of hereditary spastic paraplegia

**DOI:** 10.1242/bio.054296

**Published:** 2020-09-29

**Authors:** Bingjie Wang, You Yu, Lai Wei, Yan Zhang

**Affiliations:** State Key Laboratory of Membrane Biology, School of Life Sciences, PKU-IDG/McGovern Institute for Brain Research, Peking University, Beijing 100871, China

**Keywords:** Receptor expression-enhancing protein 1, Hereditary spastic paraplegias, Endoplasmic reticulum stress, Salubrinal

## Abstract

Hereditary spastic paraplegias (HSPs) are genetic neurodegenerative diseases. HSPs are characterized by lower-extremity weakness and spasticity. However, there is no specific clinical treatment strategy to prevent or reverse nerve degeneration in HSPs. Mutations in receptor expression-enhancing protein 1 (REEP1) are well-recognized and relatively common causes of autosomal dominant HSPs. REEP1 modifies the endoplasmic reticulum (ER) shape, and is implicated in the ER stress response. Defects in the ER stress response seem to be crucial mechanisms underlying HSP neurodegeneration. Here, we report that REEP1^−/−^ mice exhibit progressive motor deficits, along with denervation of neuromuscular junctions and increased ER stress. Moreover, marked axonal degeneration and morphological abnormalities are observed. In this study, we treated both REEP1^−/−^ and wild-type (WT) mice with salubrinal, which is a specific inhibitor of ER stress, and we observed increased nerve-muscle connections and enhanced motor functions. Our data highlight the importance of ER homeostasis in HSPs, providing new opportunities for HSP treatment.

## Abstract (precis)

**Summary:** Endoplasmic reticulum (ER) stress plays an important role in hereditary spastic paraplegias (HSPs) pathology, providing new opportunities for HSP treatment.

## INTRODUCTION

Hereditary spastic paraplegias (HSPs) are genetic neurodegenerative diseases with a prevalence of ∼1.3–9.6 in 100,000 individuals (Guglielmi, 2020). HSPs are characterized by lower-extremity weakness and spasticity and distal-end degeneration of long motor neuron axons (Blackstone et al., 2011; Harding, 1993; Tesson et al., 2015). However, no specific clinical treatment strategies are available to prevent or reverse the nerve degeneration caused by HSPs. Thus, further research into the genetic causes, pathological mechanisms, and disease progression of HSPs is urgently needed to provide clinicians with new treatment strategies. To date, more than 70 distinct genetic loci have been implicated in HSPs (Lim et al., 2015). Most types of HSPs are designated by their genetic loci (Spastic paraplegia, SPG1-78), which are numbered in order of their discovery (Blackstone, 2018; Fink, 2013; de Souza et al., 2017; Guglielmi, 2020). Autosomal dominant HSPs are the most common type and found in 75–80% of patients. Most cases (∼50%) of HSPs result from autosomal dominant mutations of just three genes: SPG3A/*ATL1*, SPG4/*SPAST*, and SPG31/*REEP1* (Park et al., 2010; Guglielmi, 2020). SPG3A is an autosomal dominant pure HSP resulting from heterozygous mutations in the gene *ATL1*. SPG4 is an autosomal-dominant HSP resulting from heterozygous mutations in the *SPAST* gene. SPG31 is an HSP caused by pathogenic variants in the *REEP1* gene and codes the receptor expression-enhancing protein-1 (REEP1), which is involved in mitochondrial and endoplasmic reticulum (ER) protein processing and transport in motor neurons in the spinal cord and brain (de Souza et al., 2017).

Mutations in REEP1 are well-recognized and relatively common causes of autosomal dominant HSPs, and are found in approximately 5% of individuals (Park et al., 2010; Guglielmi, 2020). REEP2 (the most closely related ortholog of REEP1) mutations have also been identified as a cause of SPG72 (Roda et al., 2017; Esteves et al., 2014). However, little is known about the mechanism by which REEP1 mutations lead to HSPs. The Beetz group generated an HSP mouse model by deleting exon 2 in *REEP1*, and these animals developed a gait disorder closely resembling SPG31 in humans. REEP-null mouse neurons also had defects in the ER structure (Beetz et al., 2013). Furthermore, another group found a link between alterations in the ER morphogenesis and lipid abnormalities, with important pathogenic implications for the most common forms of HSP (Renvoisé et al., 2016). REEP1-deficient mice can be used as an effective tool with which to examine HSP to study the related cellular mechanisms, pathology, and potential treatments.

Endogenous REEP1 has been detected in the brain, spinal cord, and testes (Hurt et al., 2014; Beetz et al., 2013). REEP1 was not detected in skeletal muscle, heart, colon, spleen, pancreas, kidney, liver, or lung (Hurt et al., 2014). At the subcellular level, endogenous REEP1 is associated with ER membranes (Beetz et al., 2013), whereas REEP1 was initially reported to localize in the mitochondria (Züchner et al., 2006). REEP1 is an ER resident protein. *In vitro* and *in vivo* studies have revealed that REEP1 plays a role in different ER-related pathways (Friedman et al., 2011; Beetz et al., 2013). REEP1 modifies ER shaping, and is implicated in the ER stress response (Park et al., 2010). Defects in the ER stress response seem to be crucial mechanisms underlying HSP neurodegeneration. An analysis of the function of the HSP protein showed that various proteins are involved in ER morphology, protein folding and the ER stress response, including the reticulon-2 protein, atlastin-1, spastin, REEP1, REEP2, NIPA1 neuronal protein, strumpellin protein and seipin, which cause SPG12, SPG3A, SPG4, SPG31, SPG72, SPG6, SPG8 and SPG17 when mutated, respectively (Esteves et al., 2014; Fink, 2013). In fact, recent studies have reported that REEP1 has an interesting anti-ER stress function and plays a role in promoting neuronal resistance to ER stress in animal models (Appocher et al., 2014).

What is the link between REEP1 and motor abilities and functions? To answer this question, we utilized REEP1^−/−^ mice, which were described previously (Beetz et al., 2013; Deutch et al., 2013). Herein, we report that REEP1^−/−^ mice exhibit progressive motor deficits, denervation of neuromuscular junctions, increased ER stress in the spinal cord motor neurons, marked axonal degeneration and morphological abnormalities. In this study, we aimed to elucidate the applicability and safety of salubrinal, which is a specific inhibitor of ER stress, for use in HSP treatment. We treated both REEP1^−/−^ and wild-type (WT) mice with salubrinal and observed increased nerve–muscle connections and enhanced motor functions in REEP1^−/−^ mice. Our data highlight the importance of ER homeostasis in HSPs, providing new opportunities for HSP treatment.

## RESULTS

### REEP1^−/−^ mice showed progressive motor deficits

**Fig. 1.**
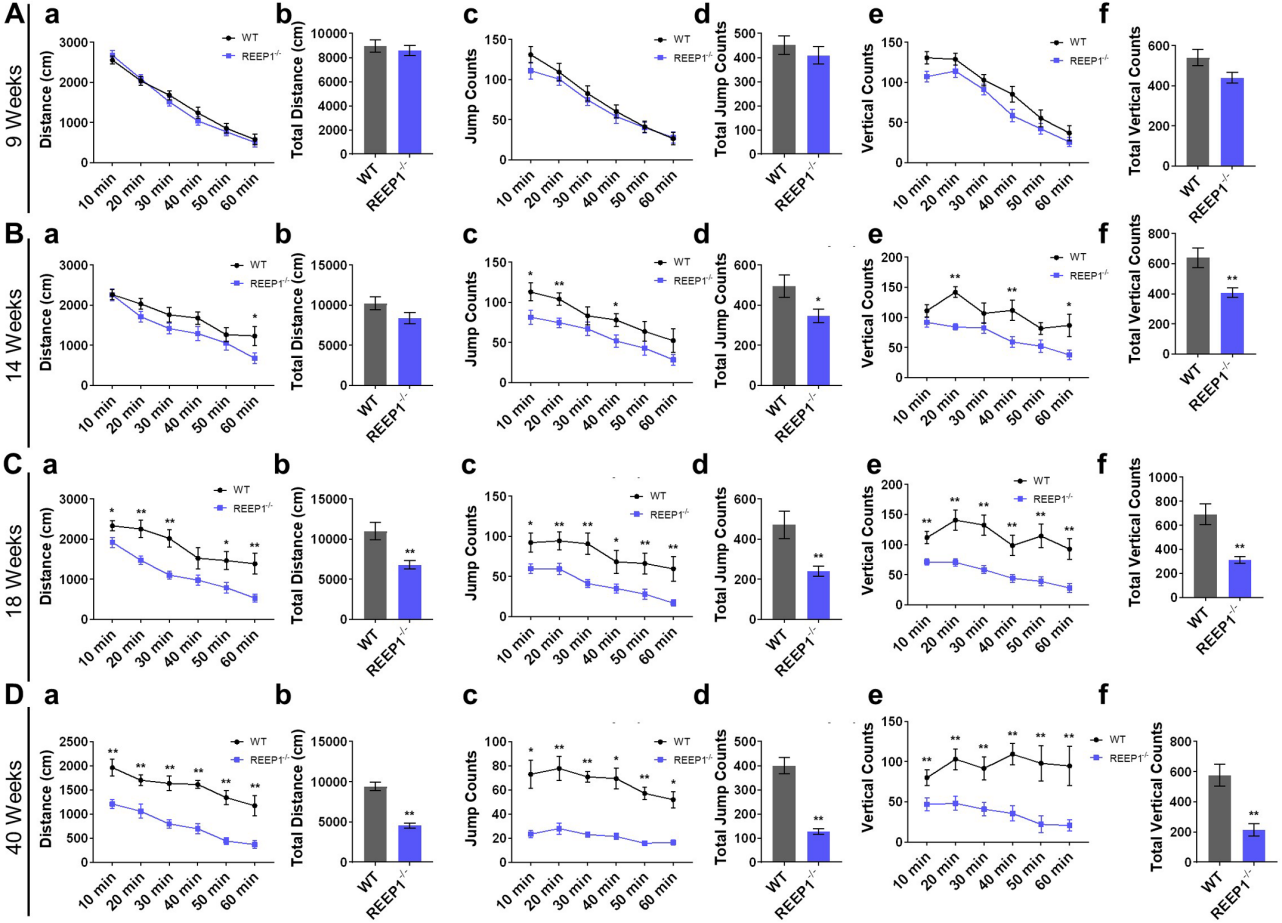
**REEP1^−/−^ mice showed progressive motor deficits in the open field test.** (A) Nine-week-old REEP1^−/−^ mice showed no difference in distance (Aa), total distance (Ab), jump counts (Ac), total jump counts (Ad), vertical counts (Ae) or total vertical counts (Af) in the open field test. WT: *n*=23, REEP1^−/−^: *n*=21. (B) Fourteen-week-old REEP1^−/−^ mice showed no difference in distance (Ba) or total distance (Bb) travelled but performed fewer jumps (Bc), total jumps (Bd), vertical movements (Be) and total vertical movements (Bf) in the open field test. WT: *n*=11, REEP1^−/−^: *n*=18. (C) Eighteen-week-old REEP1^−/−^ mice travelled a shorter distance (Ca) and a shorter total distance (Cb) and performed fewer jumps (Cc), fewer total jumps (Cd), fewer vertical movements (Ce) and fewer total vertical movements (Cf) in the open field test. WT: *n*=14, REEP1^−/−^: *n*=21. (D) Forty-week-old REEP1^−/−^ mice travelled a shorter distance (Da) and a shorter total distance (Db) and performed fewer jumps (Dc), fewer total jumps (Dd), fewer vertical movements (De) and fewer total vertical movements (Df) in the open field test. WT: *n*=25, REEP1^−/−^: *n*=21. All data are presented as the mean±s.e.m. *P<0.05, **P<0.01, ***P<0.001.

Preliminary works have reported that REEP1^−/−^ mice exhibit an abnormal gait, with the angle between their hindquarters and the ground decreasing as they grow older. Axonal degeneration of the corticospinal neurons has also been found in these mutants (Beetz et al., 2013). Therefore, we tested the motor abilities of WT and REEP1^−/−^ mice at multiple ages (9, 14, 18 and 40 weeks). In the open field test, the autonomic movement distance (Fig. 1A–D,a,b), jump counts (Fig. 1A–D,c,d), and vertical counts (Fig. 1A–D,e,f) of REEP1^−/−^ mice were lower than those of WT mice, and these deficits developed in a progressive manner.

**Fig. 2.**
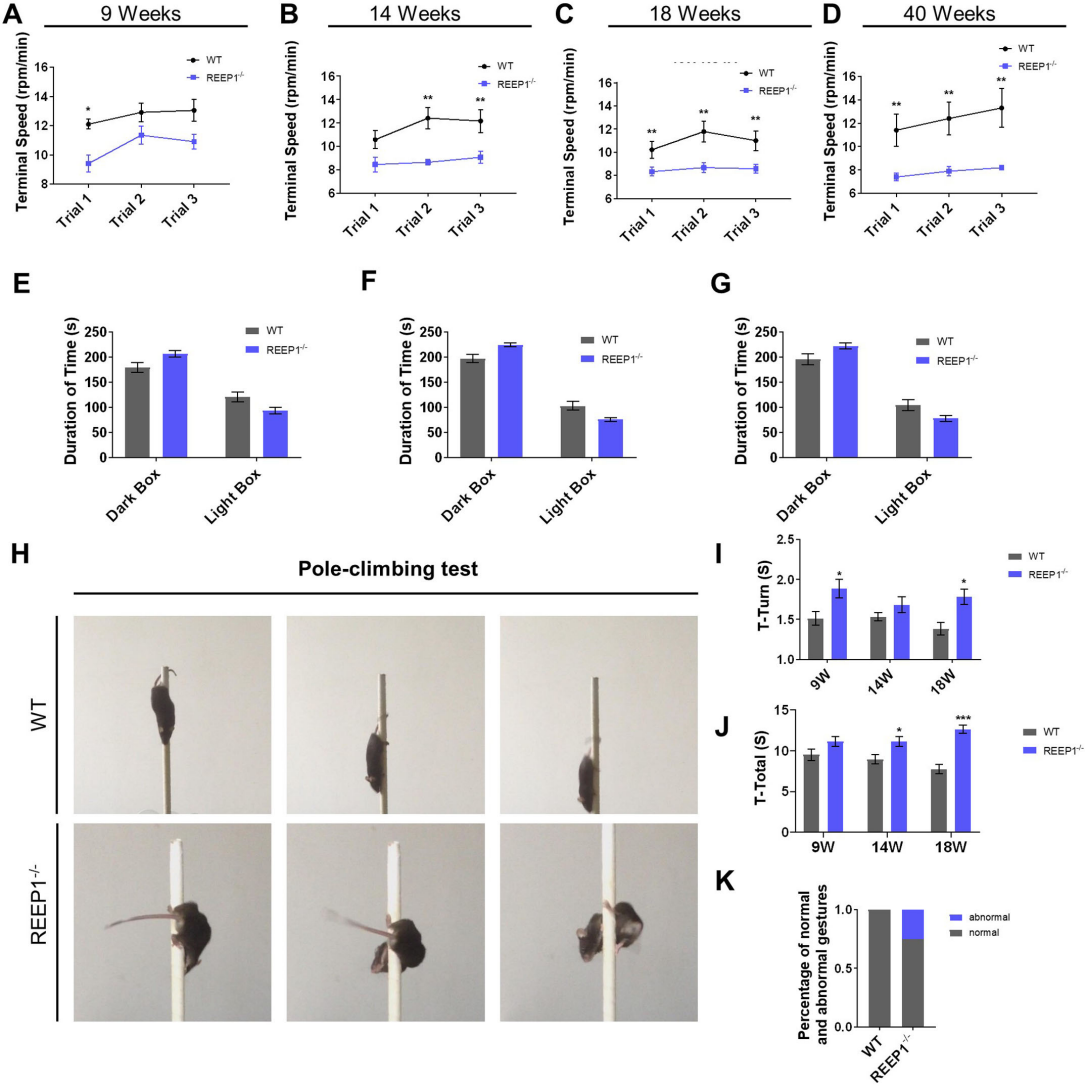
**REEP1^−/−^ mice showed progressive motor deficits in the rotarod test and pole-climbing test.** (A–D) Performance of WT and REEP1^−/−^ mice in the rotarod test at 9 weeks (WT: *n*=20, REEP1^−/−^: *n*=20), 14 weeks (WT: *n*=15, REEP1^−/−^: *n*=19), 18 weeks (WT: *n*=15, REEP1^−/−^: *n*=17) and 40 weeks (WT: *n*=10, REEP1^−/−^: *n*=10) of age. (E–G) We found no significant difference in anxiety levels between REEP1^−/−^ mice and WT mice at 9 weeks (WT: *n*=25, REEP1^−/−^: *n*=19), 14 weeks (WT: *n*=11, REEP1^−/−^: *n*=16) or 18 weeks (WT: *n*=21, REEP1^−/−^: *n*=14) of age. (H) Representative photographs of WT and REEP1^−/−^ mice performing the pole-climbing test. (I) The T-turn of 9-week-old (WT: *n*=9, REEP1^−/−^: *n*=11), 14-week-old (WT: *n*=16, REEP1^−/−^: *n*=18), and 18-week-old (WT: *n*=8, REEP1^−/−^: *n*=12) mice (two-way ANOVA: interaction, *F*_2,64_=1.223, *P*=0.3010; time, *F*_2,64_=0.7244, *P*=0.4885; genotype, *F*_1,64_=14.78, *P*=0.0003. Sidak's multiple comparisons test: WT versus REEP1^−/−^, 9-week-old: *P*=0.0338; 14-week-old: *P*=0.4477; 18-week-old: *P*=0.0411). (J) The time required for 9-week-old (WT: *n*=9, REEP1^−/−^: *n*=11), 14-week-old (WT: *n*=16, REEP1^−/−^: *n*=18), and 18-week-old (WT: *n*=8, REEP1^−/−^: *n*=12) mice to finish a trial. Eighteen-week-old REEP1^−/−^ mice took longer to finish the trial than the WT mice (two-way ANOVA: interaction, *F*_2, 64_=2.940, *P*=0.0601; time, *F*_2, 64_=0.1147, *P*=0.8918; genotype, *F*_1,64_=28.53, *P*<0.0001. Sidak's multiple comparisons test: WT versus REEP1^−/−^, 9-week-old: *P*=0.2587; 14-week-old: *P*=0.0149; 18-week-old: *P*<0.0001). (K) Statistical analysis of abnormal posture in the pole-climbing test. The proportion of REEP1^−/−^ mice that assumed an abnormal posture was significantly higher than that of WT mice that assumed an abnormal posture. All data are presented as the mean±s.e.m. *P<0.05, **P<0.01, ***P<0.001.

Impaired autonomic movement ability may be the result of compromised motor abilities or anxiety. We further assessed the exercise capacity and anxiety levels of the mice separately. REEP1^−/−^ mice tended to stay on the rotarod for a shorter period of time compared to their WT littermates and had a lower terminal speed when they fell off the rod (Fig. 2A–D). Moreover, no significant differences were found in the light/dark box transition test (Fig. 2E–G). Therefore, we assume that impairments in motor abilities rather than anxiety (Fig. 2E–G) are responsible for the observed deficits (Figs 1 and 2A–D). We used the pole-climbing test to further evaluate the motor abilities. A group of REEP1^−/−^ mice showed abnormal behavior during the test the mice showed a spiral downward crawling posture (Fig. 2H,K). Although there was no statistical significance in the T-turn (Fig. 2I), mutant mice required a greater amount of time compared to WT littermates to finish each trial (Fig. 2J). It is worth mentioning that 40-week-old REEP1^−/−^ mice were not able to turn on the rod at all. These observations are consistent with the features of SPG31. SPG31 patients usually show symptoms at an early age, but the disease progresses rather slowly. Based on the results of the abovementioned behavioral tests, which showed that 40-week-old mutants displayed dramatic motor deficits, we used 40-week-old mice for our subsequent studies.

### Denervation of neuromuscular junctions in REEP1^−/−^ mice

**Fig. 3.**
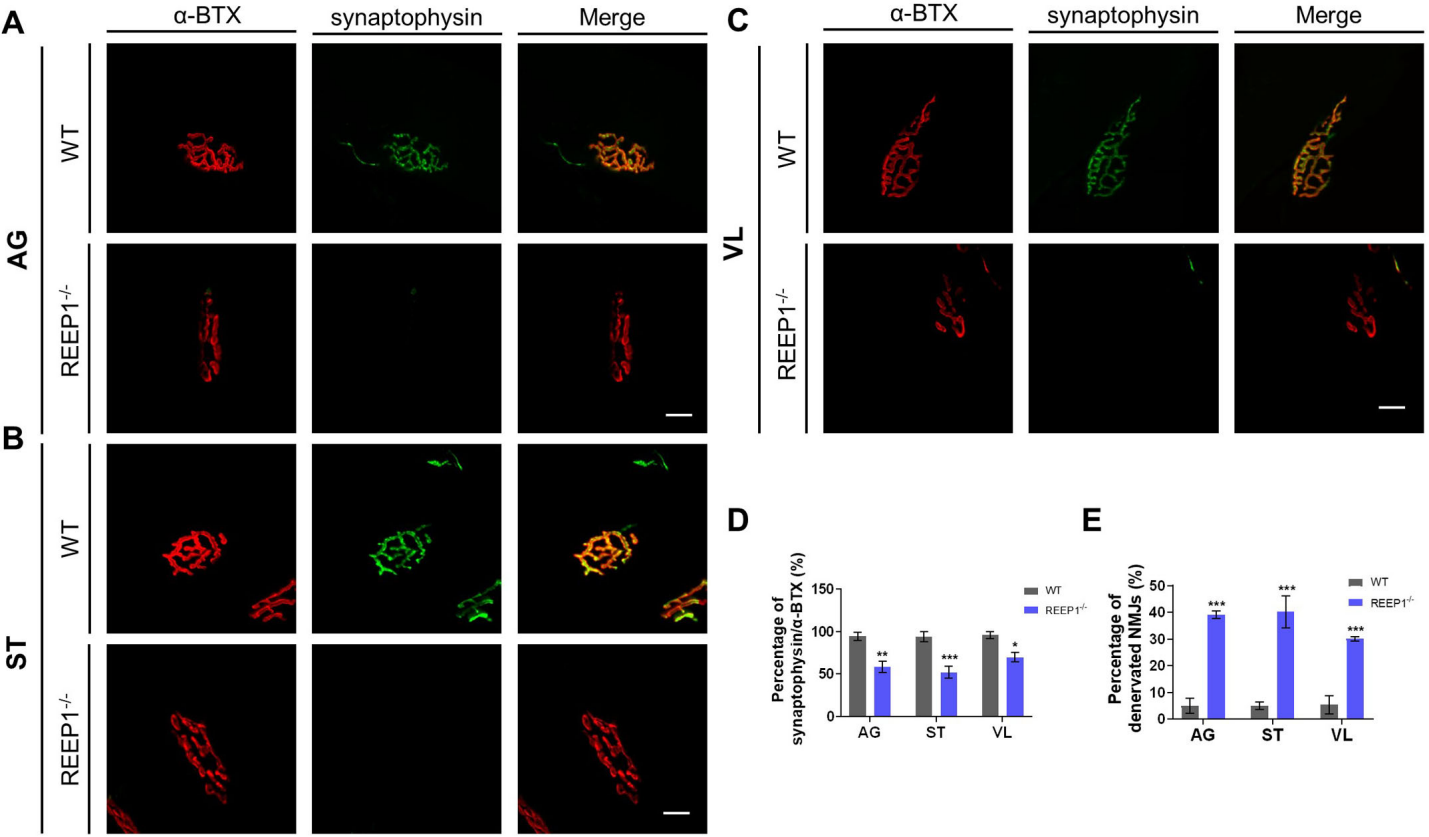
**Denervation of NMJs in REEP1^−/−^ mice.** (A) Co-staining for synaptophysin and α-BTX to quantify the innervation rate of NMJs in the anterior gracilis (AG). Scale bar: 5 μm. (B) Co-staining synaptophysin and α-BTX to quantify the innervation rate of NMJs in the semitendinosus (ST). Scale bar: 5 μm. (C) Co-staining for synaptophysin and α-BTX to quantify the innervation rate of NMJs in the vastus lateralis (VL). Scale bar: 5 μm. (D) The area of synaptophysin/α-BTX colocalization was significantly reduced in all three kinds of muscles in REEP1^−/−^ mice compared to WT mice (two-way RM ANOVA: interaction, *F*_2,8_=34.30, *P*=0.0001; muscle, *F*_2.8_=52.10, *P*<0.0001; genotype, *F*_1,4_=18.74, *P*=0.0124; subjects: *F*_4,8_=99.96, *P*<0.0001. Sidak's multiple comparisons test: WT versus REEP1^−/−^, AG: *P*=0.0024; ST: *P*=0.0007; VL: *P*=0.0221). (E) The percentage of denervated NMJs was higher in REEP1^−/−^ mice than in WT mice (two-way RM ANOVA: interaction, *F*_2,8_=2.104, *P*=0.1844; muscle, *F*_2.8_=1.833, *P*=0.2211; genotype, *F*_1,4_=101.1, *P*=0.0006; subjects: *F*_4,8_=1.849, *P*=0.2129. Sidak's multiple comparisons test: WT versus REEP1^−/−^, AG: p *P*<0.0001; ST: p *P*<0.0001; VL: p *P*=0.0004). Data from three independent experiments. All data are presented as the mean±s.e.m. *P<0.05, **P<0.01, ***P<0.001.

Body movements depend on the upper and spinal cord motor neurons. Researchers have reported degeneration in the upper motor neurons in HSPs (Beetz et al., 2013), so we focused on spinal cord motor neurons. Since we found progressive motor dysfunctions in REEP1^−/−^ mice, we speculated that there might be reductions in muscular force among the 40-week-old mutants. We co-stained for synaptophysin and α-bungarotoxin (α-BTX) to quantify the innervation rate of neuromuscular junctions (NMJs). Denervation is a common type of NMJ injury, and synaptophysin and α-BTX are typically used as markers for studying the denervation of NMJs (Saxena et al., 2009). When the colocalization rate was greater than 80%, we considered the muscle to be fully innervated, and when colocalization rate was lower than 30%, we considered the muscle to be denervated (Saxena et al., 2009). Of the 39 extensively categorized kinds of hind limb muscles in the mouse (Charles et al., 2016), we chose the anterior gracilis (AG), semitendinosus (ST), and vastus lateralis (VL) muscles for the experiments, as these muscles are responsible for the movement of the hindquarters and knees. The AG is a hip adductor; the ST is a hip extensor; and the VL is a knee extensor (Charles et al., 2016). It was found that the area of synaptophysin/α-BTX colocalization was significantly reduced in all three kinds of muscles in REEP1^−/−^ mice compared to WT mice, and accordingly, the percentage of denervated NMJs was also much higher in the mutant mice than in the WT mice (Fig. 3A–E).

**Fig. 4.**
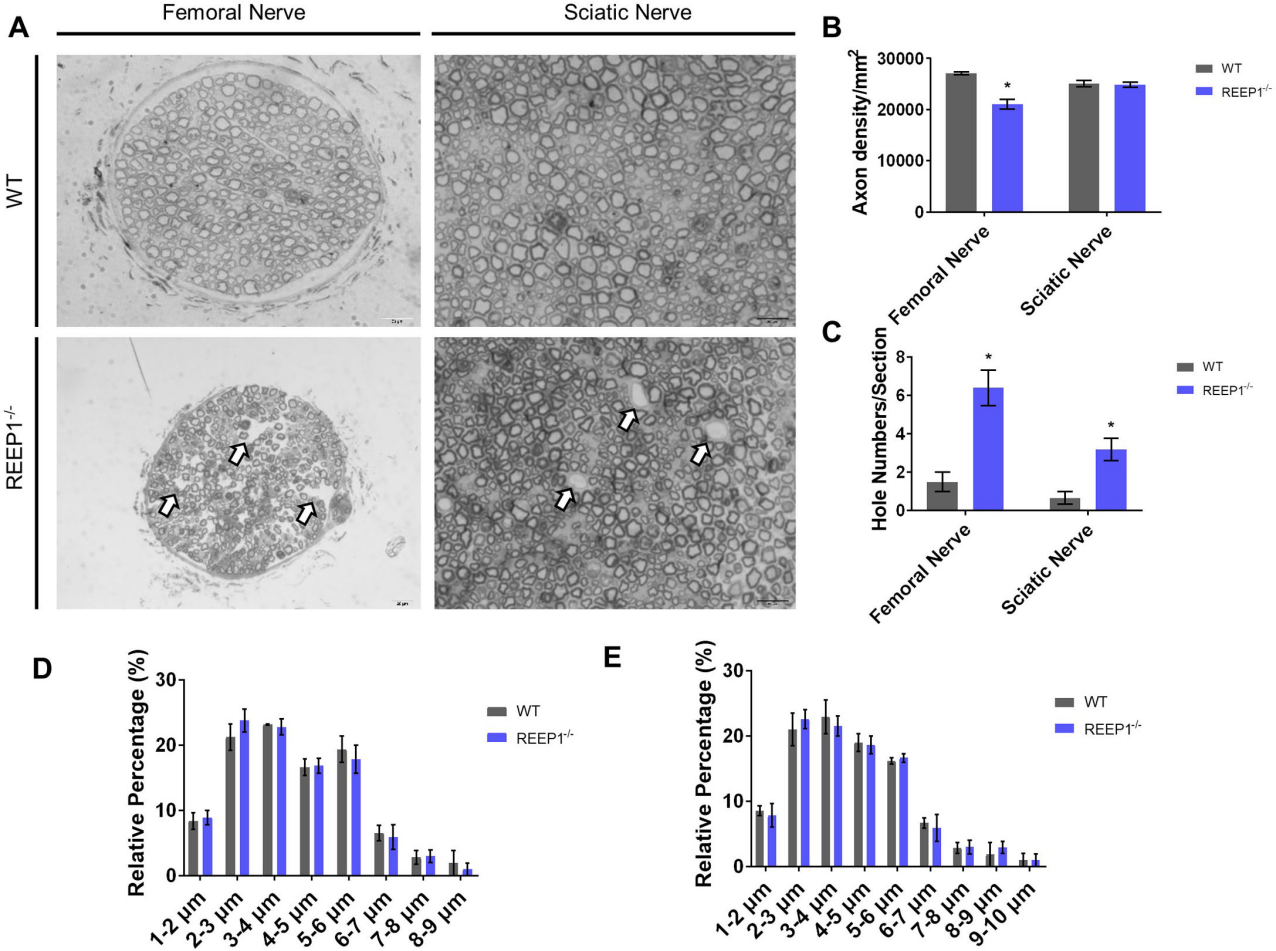
**REEP1^−/−^ mice exhibited axonal degeneration.** (A) Representative TEM images of femoral nerves and sciatic nerves of WT and REEP1^−/−^ mice. Arrows point to axonal holes. Scale bars: 20 μm. (B) We found that the axonal density in the femoral nerve was significantly lower in REEP1^−/−^ mice than in WT mice (*P*=0.0251), while the sciatic nerve density did not show obvious changes (*P*=0.8934). (C) The number of axonal holes was significantly increased in the femoral nerves and sciatic nerves of REEP1^−/−^ mice compared to WT mice (p_FN_=0.9143, p_SN_=0.9237). (D) Distribution patterns of the femoral nerves (two-way RM ANOVA: interaction, *F*_7,28_=0.1311, *P*=0.9951; width, *F*_7,28_=57.97, *P*<0.0001; genotype, *F*_1,4_=1.087, *P*=0.3560; subjects: *F*_4,28_=0.0001, *P*>0.9999). (E) Distribution patterns of the sciatic nerves (two-way RM ANOVA: interaction, *F*_7,28_=0.1771, *P*=0.9879; width, *F*_7,28_=121.8, *P*<0.0001; genotype, *F*_1,4_=0.9863, *P*=0.3769; subjects: *F*_4,28_=0.0001, *P*>0.9999). *n*=20 from four mice per group. All data are presented as the mean±s.e.m. *P<0.05, **P<0.01, ***P<0.001.

As the muscles of the mutants remained intact (data not shown), the denervation may have been due to axonal degeneration of the spinal cord motor neurons. We collected cross-sections of sciatic nerve and femoral nerve fibers, which drive the movement of the hindquarters. We stained the sections with Toluidine Blue to label myelin sheaths and imaged them under a TEM. Under the TEM, stained the myelin sheaths appeared dark blue, while the interior of the axons remained transparent. Marked axonal degeneration and morphological abnormalities were observed (Fig. 4A). The density of the femoral nerve axons was reduced significantly, and holes were observed in the nerve fibers. Although the axonal density of the sciatic nerve remained unchanged, holes were observed in this nerve as well (Fig. 4B,C). This phenomenon has also been reported in the spinal cords of amyotrophic lateral sclerosis (ALS) mice (King et al., 2012) and corticospinal tract of REEP1^−/−^ mice (Beetz et al., 2013). We eliminated the possibility of a change in the axonal length distribution by calculating the relative percentages of axons of different lengths. The distribution patterns remained the same in both the sciatic and femoral nerves (Fig. 4D,E). Taken together, the results demonstrated that axonal degeneration of the spinal cord motor neurons in REEP1^−/−^ mice led to the reduced innervation of these muscles, which further caused the loss of muscular force in REEP1^−/−^ mice.

### REEP1^−/−^ mice showed increased ER stress in spinal cord motor neurons

As previously reported, overexpressing the human REEP1 gene in *Drosophila* can promote the resistance of neurons to ER stress (Appocher et al., 2014). Several proteins encoded by HSP genes in addition to REEP1, including reticulon-2 protein, atlastin-1, spastin, REEP1, REEP2, the NIPA1 neuronal protein, strumpellin protein and seipin, have been reported to be associated with the ER morphology and ER stress response (Esteves et al., 2014; Fink, 2013). ER stress has also been found in an ALS mouse model, in which stress induces axonal degeneration, muscle denervation and apoptosis of motor neurons (Saxena et al., 2009). REEP1^−/−^ mice neurons also had defects in the ER structure (Beetz et al., 2013). Hence, it is of great value to examine the level of ER stress and subsequent axonal degeneration of motor neurons in REEP1^−/−^ mice.

**Fig. 5.**
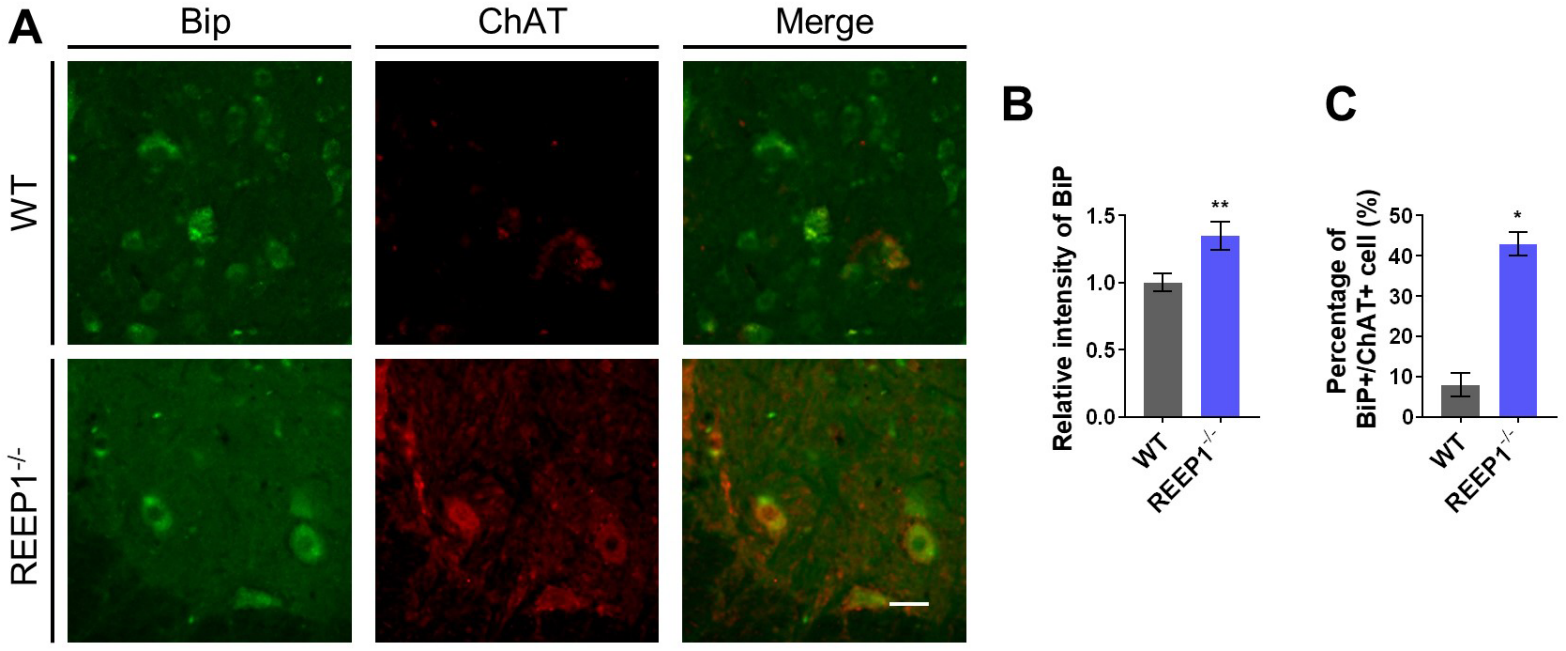
**REEP1^−/−^ mice motor neurons showed increased ER stress.** (A) Co-stained Ig binding protein (Bip) and ChAT to quantify the level of ER stress in WT and REEP1^−/−^ mice. ChAT (red), Bip (green). Scale bar: 50 μm. (B) Expression level of BiP significantly increased in REEP1^−/−^ mice motor neurons. (C) Percentage of stressed motor neurons significantly increased in REEP1^−/−^ mice motor neurons. WT: *n*=60, REEP1^−/−^: *n*=63. All data are presented as the mean±s.e.m. *P<0.05, **P<0.01, ***P<0.001.

First, we verified the expression of REEP1 in the brain and muscle. We found REEP1 expression in the brain instead of muscle (Fig. S1A). It is also reported that REEP1 was strongly expressed in the lower motor neurons (Beetz et al., 2012). Similar results were also found in the spinal cord motor neurons of WT mice (Fig. S1B). To demonstrate our hypothesis, we used a binding immunoglobulin protein (BiP) as a specific marker to test the level of ER stress in the spinal cord motor neurons. Compared to WT mice, REEP1^−/−^ mice exhibited a slight increase in BiP expression at the age of 40 weeks (Fig. 5A,B). Forty-three percent of the tested motor neurons in the REEP1^−/−^ mice showed increased levels of ER stress, whereas only 8% of motor neurons in the WT mice showed such an increase (Fig. 5C).

### Salubrinal increased motor functions in REEP1^−/−^ mice

**Fig. 6.**
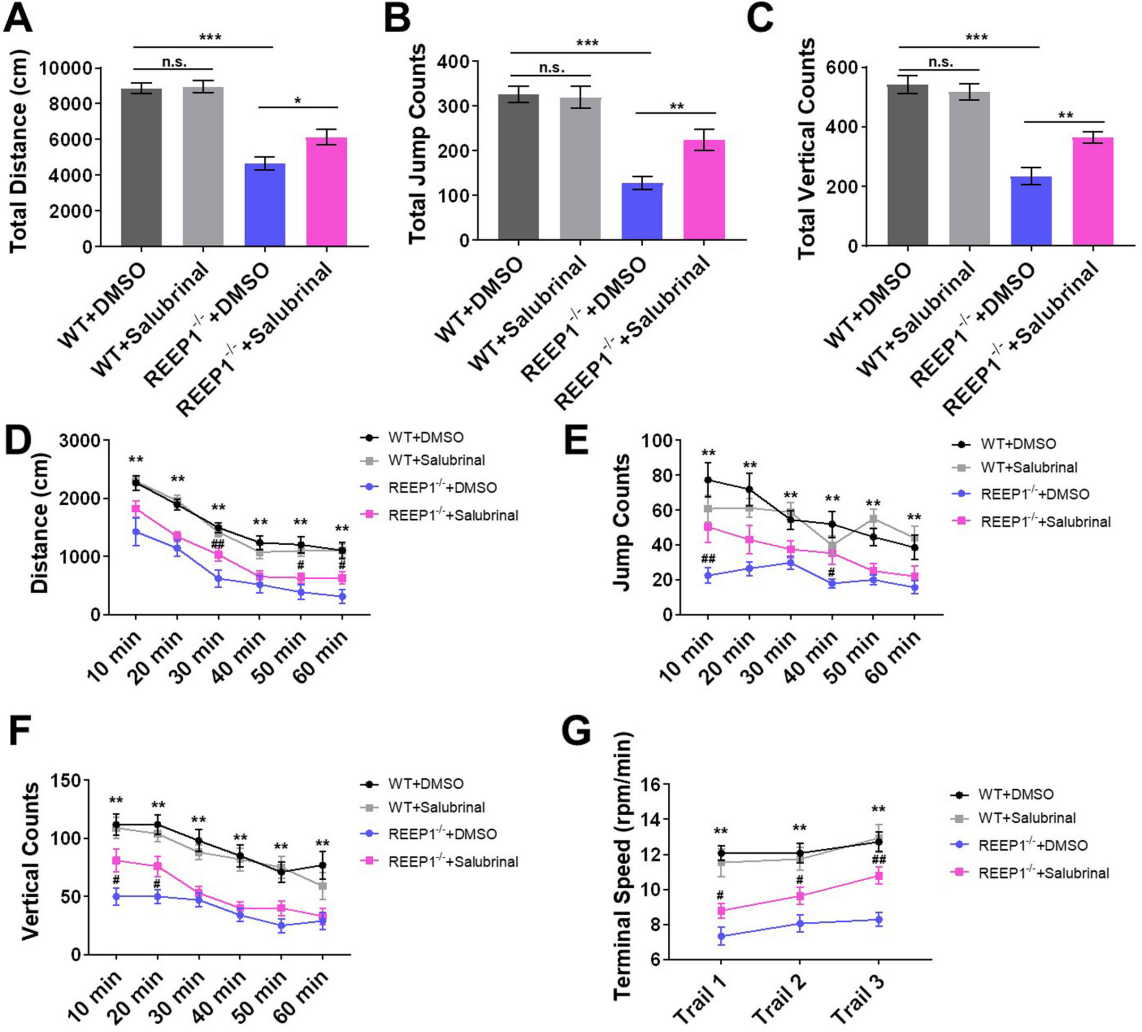
**Salubrinal increased motor functions in REEP1^−/−^ mice.** (A) Compared to no treatment, the salubrinal treatment increased the total distance in REEP1^−/−^ mice (two-way ANOVA: interaction, *F*_1, 62_=3.109, *P*=0.0828; treatment, *F*_1,62_=4.121, *P*=0.0467; genotype, *F*_1, 62_=83.25, *P*<0.0001. Sidak's multiple comparisons test: WT+DMSO versus WT+salubrinal, *P*=0.9980; REEP1^−/−^+DMSO versus REEP1^−/−^+salubrinal, *P*=0.0286; WT+DMSO versus REEP1^−/−^+DMSO, *P*<0.0001). (B) Compared to no treatment, the salubrinal treatment increased the total jump counts in REEP1^−/−^ mice (two-way ANOVA: interaction, *F*_1, 62_=5.957, *P*=0.0175; treatment, *F*_1,62_=4.625, *P*=0.0354; genotype, *F*_1, 62_=49.46, *P*<0.0001. Sidak's multiple comparisons test: WT+DMSO versus WT+salubrinal, *P*=0.9974; REEP1^−/−^+DMSO versus REEP1^−/−^+salubrinal, *P*=0.0052; WT+DMSO versus REEP1^−/−^+DMSO, *P*<0.0001). (C) Compared to no treatment, the salubrinal treatment increased the total vertical counts in REEP1^−/−^ mice (two-way ANOVA: interaction, *F*_1,62_=9.042, *P*=0.0038; treatment, *F*_1,62_=4.379, *P*=0.0405; genotype, *F*_1,62_=81.79, *P*<0.0001. Sidak's multiple comparisons test: WT+DMSO versus WT+salubrinal, *P*=0.9284; REEP1^−/−^+DMSO versus REEP1^−/−^+salubrinal, *P*=0.0016; WT+DMSO versus REEP1^−/−^+DMSO, *P*<0.0001). (D–F) Compared to no treatment, the salubrinal treatment increased the autonomic movement distance, jump counts and vertical counts. WT+DMSO: *n*=14, WT+salubrinal: *n*=15, REEP1^−/−^+DMSO: *n*=18, REEP1^−/−^+salubrinal: *n*=19. (G) REEP1^−/−^ mice performed better in the rotarod test after salubrinal treatment. WT+DMSO: *n*=14, WT+salubrinal: *n*=15, REEP1^−/−^+DMSO: *n*=18, REEP1^−/−^+salubrinal: *n*=19. All data are presented as the mean±s.e.m. *P<0.05, **P<0.01, ***P<0.001.

We treated both the REEP1^−/−^ and WT mice with salubrinal, which is a specific inhibitor of ER stress that promotes stress resistance by inhibiting elF2α phosphatase (Wang et al., 2019). Various studies linking HSPs and ER stress have inspired us to study ER stress inhibitors as potential means by which to alleviate the phenotype of HSPs and indicated that salubrinal treatment should be able to relieve the reductions in motor function observed in REEP1^−/−^ mice. Salubrinal treatment increased the autonomic movement distance (Fig. 6A,D), jump counts (Fig. 6B,E) and rear counts (Fig. 6C,F) in the open field test in REEP1^−/−^ mice. Additionally, the mutants performed better in the rotarod test after salubrinal treatment (Fig. 6G). At the same time, we found that salubrinal treatment did not affect the WT mice performance in the open field test or the rotarod test.

**Fig. 7.**
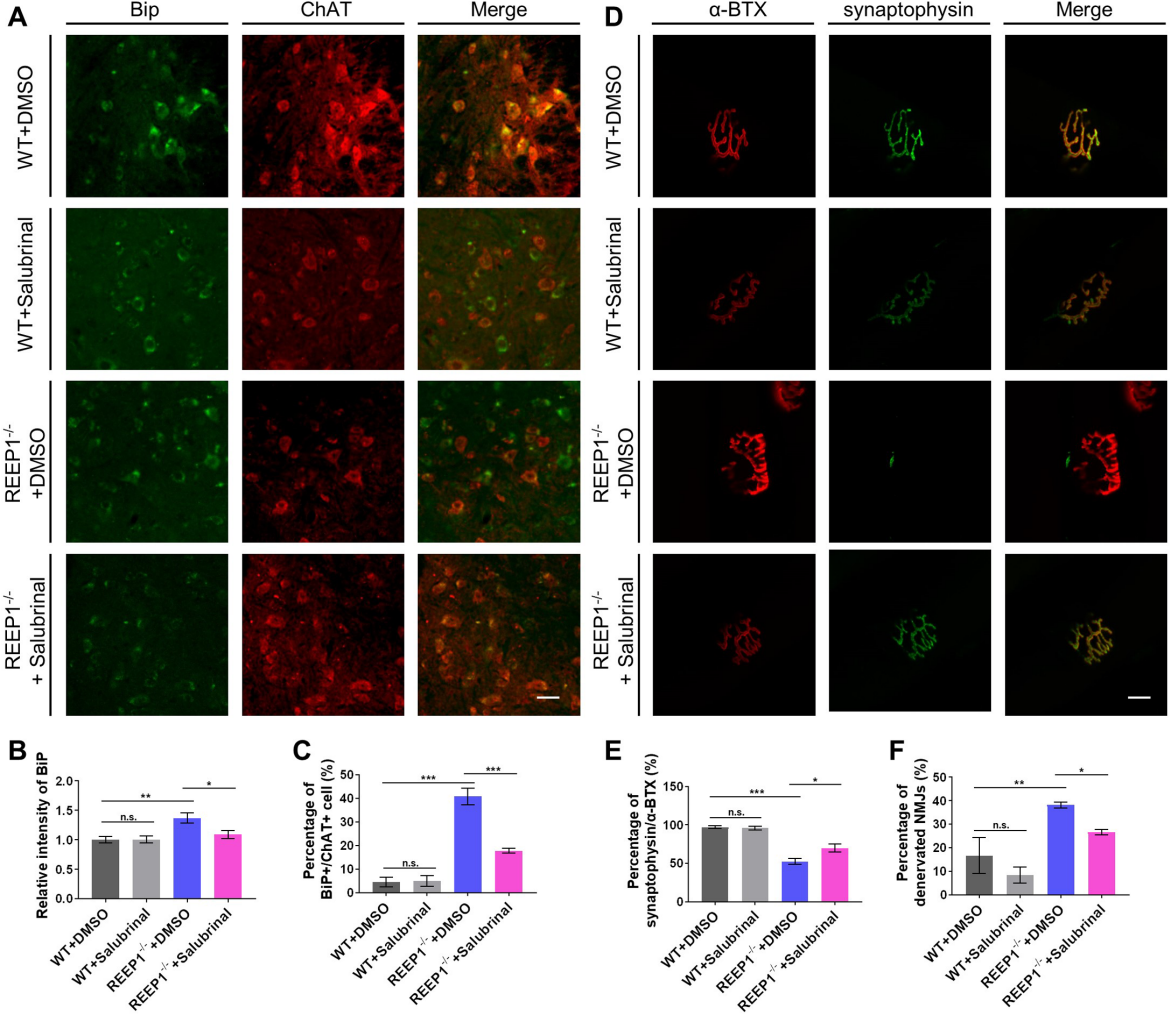
**Salubrinal increased REEP1^−/−^ mice motor functions by improving NMJs and ER stress.** (A) Co-staining for BiP and ChAT to quantify the level of ER stress in WT and REEP1^−/−^ mice treated with salubrinal or DMSO. ChAT (red), BiP (green). Scale bar: 50 μm. (B) The expression level of BiP was significantly decreased in the motor neurons of salubrinal-treated REEP1^−/−^ mice compared to DMSO-treated REEP1^−/−^ mice (two-way ANOVA: interaction, *F*_1,225_=4.112, *P*=0.0438; treatment, *F*_1,225_=3.906, *P*=0.0493; genotype, *F*_1,225_=10.31, *P*=0.0015. Sidak's multiple comparisons test: WT+DMSO versus WT+salubrinal, *P*>0.9999; REEP1^−/−^+DMSO versus REEP1^−/−^+salubrinal, *P*=0.02531; WT+DMSO versus REEP1^−/−^+DMSO, *P*=0.0017). WT+DMSO: *n*=55, WT+salubrinal: *n*=59, REEP1^−/−^+DMSO: *n*=58, REEP1^−/−^+salubrinal: *n*=57. (C) Percentage of stressed motor neurons was significantly decreased in the motor neurons of salubrinal-treated REEP1^−/−^ mice compared to untreated REEP1^−/−^ mice (two-way ANOVA: interaction, *F*_1,12_=23.80, *P*=0.004; treatment, *F*_1,12_=21.81, *P*=0.0005; genotype, *F*_1,12_=103.5, *P*<0.0001. Sidak's multiple comparisons test: WT+DMSO versus WT+salubrinal, *P*>0.9999; REEP1^−/−^+DMSO versus REEP1^−/−^+salubrinal, *P*=0.0001; WT+DMSO versus REEP1^−/−^+DMSO, *P*<0.0001). Data from four independent experiments. (D) Co-staining for synaptophysin and α-BTX to quantify the innervation rate of NMJs in the ST. Scale bar: 5 μm. (E) The area of synaptophysin/α-BTX colocalization was significantly increased in salubrinal-treated REEP1^−/−^ mice compared to untreated REEP1^−/−^ mice (two-way ANOVA: interaction, *F*_1,8_=7.306, *P*=0.0265; treatment, *F*_1,8_=5.688, *P*=0.0442; genotype, *F*_1,8_=105.5, *P*<0.0001. Sidak's multiple comparisons test: WT+DMSO versus WT+salubrinal, *P*=0.9952; REEP1^−/−^+DMSO versus REEP1^−/−^+salubrinal, *P*=0.0285; WT+DMSO versus REEP1^−/−^+DMSO, *P*<0.0001). Data from three independent experiments, with each experiment including four mice. (F) Percentage of denervated NMJs was lower in the salubrinal-treated REEP1^−/−^ mice than in the untreated REEP1^−/−^ mice (two-way ANOVA: interaction, *F*_1,8_=0.1494, *P*=0.7092; treatment, *F*_1,8_=5.458, *P*=0.0477; genotype, *F*_1,8_=21.56, *P*=0.0017. Sidak's multiple comparisons test: WT+DMSO versus WT+salubrinal, *P*=0.5444; REEP1^−/−^+DMSO versus REEP1^−/−^+salubrinal, *P*=0.0050; WT+DMSO versus REEP1^−/−^+DMSO, *P*=0.0305). Data from three independent experiments, each experiment has four mice. All data are presented as the mean±s.e.m.

To verify the effect of salubrinal application, we examined the ER stress level of WT and REEP1^−/−^ mice with or without salubrinal treatment. In the previous results (Fig. 3D,E), ST was found to have the largest difference, whether the percentage of synaptophysin/α-BTX colocalization (51.97%±7.07) or the percentage of denervated NMJs (40.19%±6.03) among the three muscles, so the ST muscle was chosen for NMJ analysis upon salubrinal treatment. The percentage of synaptophysin/α-BTX colocalization and the percentage of denervated NMJs were higher in the REEP1^−/−^ mice than in the WT mice. Therefore, we sought to determine whether salubrinal treatment can rescue denervated NMJs observed in REEP1^−/−^ mice. We treated both the REEP1^−/−^ and WT mice with salubrinal. Compared to those in the DMSO-treated group, the expression level of BiP and the percentage of stressed motor neurons in the ventricornu were significantly decreased in REEP1-null mice in the salubrinal-treated group after 4 weeks (Fig. 7A–C). Salubrinal treatment also increased the nerve–muscle connections in the ST in REEP1^−/−^ mice, which previously showed considerable denervation (Fig. 7D–F). Consistent with the behavioral results, salubrinal treatment did not affect the ER stress level or NMJ of WT mice, which implies the possibility of salubrinal as a promising therapeutic drug in HSPs.

## DISCUSSION

Although the genes responsible for most HSPs have been identified, effective treatments for the disease are still lacking. Therefore, it is of great value to more deeply investigate the underlying functions of the *REEP1* gene and the mechanisms underlying autosomal dominant HSPs to identify new treatment strategies for this disease. Our study demonstrated axonal degeneration in spinal cord neurons and denervation of hindquarter muscles in REEP1^−/−^ mice, which together induced motor deficits in a progressive manner. Clinically, HSPs are characterized by varying degrees of lower extremity weakness and spasticity, different ages of symptom onset, and variable degrees of progression (Fink, 2013). SPG31 is an early-onset autosomal dominant neurodegenerative disease that starts from childhood to adulthood ages and can also lead to spastic paraparesis and amyotrophy. The characteristics of REEP1^−/−^ mice reflect the phenotype of SPG31.

We investigated the nerve damage in this mouse model. REEP1 is mainly located in the corticospinal neurons that exhibit axonal damage in related HSPs (Beetz et al., 2013). Nonetheless, there has been little discussion of how spinal motor neurons contribute to the mechanisms underlying SPG31. Marked axonal degeneration and morphology abnormalities were observed in the sciatic nerves and femoral nerves of REEP1^−/−^ mice. Furthermore, denervation of NMJs was observed in these mice. It can thus be deduced from these results that axonal degeneration induced NMJ denervation, which led to decreased motor function in REEP1^−/−^ mice.

As mentioned earlier, REEP1 plays vital roles in regulating ER shaping and the ER stress response (Park et al., 2010). In humans, the REEP superfamily contains six members: REEP1-6. All REEPs are membrane-bound ER proteins harboring hydrophobic hairpin domains (Roda et al., 2017). REEP1 and its most closely related ortholog REEP2 are preferentially expressed in neuronal tissues (Hurt et al., 2014). SPG31 is a HSP caused by pathogenic variants of the *REEP1* gene. Furthermore, REEP2 (the most closely-related ortholog to REEP1) mutations have also been identified as a cause of SPG72 (Roda et al., 2017; Esteves et al., 2014). We found increased ER stress levels in spinal cord motor neurons. Renvoisé et al. found a link between alterations in the ER morphogenesis and lipid abnormalities (Renvoisé et al., 2016), which may have been one of the causes of the observed denervation. To the best of our knowledge, our work is the first to describe ER stress in spinal cord motor neurons, and we believe that more attention should be paid to this process in future studies on REEP1 and other types of HSPs. Additionally, proteins such as atlastin and spastin interact with REEP1 in the ER and collectively regulate the ER morphology (Park et al., 2010).

Various studies linking HSPs and ER stress have provided us with inspiration for studying ER stress inhibitors as potential means by which to alleviate the phenotype of HSPs. Treatment with anti-ER stress agents (such as salubrinal) provides enhanced neuroprotective effects (Anuncibay-Soto et al., 2018). Therefore, we utilized REEP1^−/−^ mice to determine whether salubrinal is able to rescue locomotor and cellular defects. Treatment with this ER stress inhibitor efficiently relieved motor deficits in REEP1^−/−^ mutants. In addition, salubrinal treatment increased the nerve-muscle connections in the ST. We showed that the inhibition of ER stress might be key to alleviating the phenotypic manifestations of HSPs.

There is no effective treatment to prevent gait impairment in HSP, although several medicines are currently in clinical use, for example Lioresal, Dantrolene and Tizanidine. Lioresal, which is a GABA derivative, inhibits reflexive muscle contraction by blocking the release of excitatory neurotransmitters via interference with voltage gated calcium channels (Lake and Shah, 2019). Dantrolene is an inhibitor of calcium release from the ryanodine-sensitive ER stores and is an attractive drug for treating or preventing neuronal injury (Wen et al., 2015). Tizanidine, which is an imidazoline derivative, is a central acting noradrenergic alpha-2 receptor agonist that results in impairment of the release of excitatory amino acids such as glutamate and aspartate from spinal interneurons and increases the presynaptic inhibition of motor neurons (Ghanavatian and Derian, 2020). However, these medicines present significant complications, such as significant systemic adverse effects, drug withdrawal, catheter infection, drug overdose, failure, etc. (Hedera, 1993; Lake and Shah, 2019; Fink, 2013). A number of HSP gene products are involved in the ER morphogenesis and stress response and in mediating contacts with multiple organelles, such as mitochondria, endosome and peroxisomes (Chang et al., 2019; Lim et al., 2015; Allison et al., 2017). These effects may cause the different HSP phenotypes. ER abnormalities represent a pathology of HSPs, which implies that the treatment of ER stress may be beneficial as a generalized therapeutic approach for HSPs. Salubrinal selectively inhibits eIF2α dephosphorylation and protects other cells against endoplasmic reticulum stress-mediated apoptosis; however, it does not target specific tissues or cells. In the current study and previous study (Rani et al., 2017), no evidence of non-specific toxicity in terms of weight gain, physical activity or survival was observed due to the administration of salubrinal (0.5 mg/kg); however, studies have found that high doses of salubrinal can indeed cause problems with other types of cells and tissues. The administration of high doses of salubrinal significantly increased the cleaved caspase-12 level, thus promoting ER stress-dependent apoptotic signaling in the cortex (Gao et al., 2013). Moreover, excessive eIF2α phosphorylation (caused by a high dose of salubrinal) is poorly tolerated by β-cells and exacerbates free fatty acid-induced apoptosis (Cnop et al., 2007). When designing drugs for use in HSP therapy, the dosage and method of administration must be considered, and local administration and sustained-release administration may be more appropriate. Furthermore, the application of low-dose salubrinal in WT mice did not produce any significant changes in the exercise capacity and other aspects of the mice, implying the possibility of long-term application of salubrinal to prevent further axon degeneration in early-stage HSP patients.

Taken together, our findings provide new perspectives for HSP treatment. Peripheral neural muscular junctions, especially those of the hindquarters rather than the central nervous system might be promising new drug delivery targets. Additionally, ER stress may be used as an index of and a target for treating autosomal dominant HSPs. Using our accumulating knowledge of the processes that occur in the ER of motor neurons, we hope to obtain a much more comprehensive understanding of HSPs and to identify more effective clinical treatments for HSP patients.

## MATERIALS AND METHODS

### Animals

REEP1^−/−^ mice (C57BL/6 background) were generously provided by Dr Christian A. Hübner, Friedrich-Schiller University. Detailed information about REEP1^−/−^ mice was provided previously (Beetz et al., 2013). All animal studies were conducted in accordance with the Guide for the Care and Use of Laboratory Animals (8th edition) and approved by the Institutional Animal Care and Use Committee of Peking University. The laboratory approval number of the Association for Assessment and Accreditation of Laboratory Animal Care (AAALAC)-approved Animal Facility at Peking University Laboratory Animal Center (LAC-PKU) IACUC was LSC-ZhangY-1. The WT front primer was: CTGCAGGCTTATATTTGGCACCCTTTATCCTGAATATTATTCATACAAGG; the WT reverse primer was: CCCGGGGATATCGGCGCGCCTGAGGGAACTGGCCAGAGAG; the REEP1^−/−^ mice front primer was: TTAAAAATACCTATTAGGCTGTG; and the REEP1^−/−^ mice reverse primer was: GGAAGAAGGTGGTCTGTG. The WT amplicon length was 358 bp, and the mutant amplicon length was 163 bp.

### Open field

The apparatus had grey Plexiglas sides and a 40×40×30-cm floor. The test was initiated by placing a mouse in the center of the apparatus. In the next 60 min, the mouse activity track was recorded by the camera. The results were analyzed with Noldus Observer software (Ethovision 11.0).

### Rotarod test

The rotarod test was conducted over 2 days. On the first day, the mice were placed on a rotating rod (MED, ENV-575A, USA, the lane width was 50 mm, and the rod diameter was 30 mm) that rotated at a speed of 4 rpm for 5 min to allow the mice to learn to walk on the wheel. After the mice were trained, the speed was increased from 4 rpm to 40 rpm in even intervals within 5 min. The mice were trained three times with an interval of 30 min each time. The mice were tested the next day. Each animal underwent three trials. The length of time that the mice managed to remain on the rod and the speed at which they fell off the apparatus were recorded. The average of these measurements over three trials were used for further analysis.

### Pole climbing test

Each mouse was trained for two consecutive trials and then underwent a successive experimental trial. Each mouse was placed on the top of a vertical rod (height=60 cm, diameter=10 mm), and the time required for the mouse to reach the bottom (T-total) was recorded. The T-turn (the time required for the mouse to turn to a head-down position on the rod) was also recorded.

### Light/dark transition test

The light/dark transition test was conducted as previously described (Matsuo et al., 2010). The apparatus used for the light/dark transition test consisted of a cage (21 cm×42 cm×25 cm) divided into two sections of equal size by a partition containing a door (Med Associates). One chamber was brightly illuminated, whereas the other chamber was dark. With the door open, each mouse was placed in the dark side and allowed to move freely between the two chambers for 5 min. The time spent on each side was recorded automatically.

### Immunostaining

Mice were anaesthetized and perfused with 4% PFA. Tissues were collected from both WT and REEP1^−/−^ mice and postfixed with 4% PFA for 4 h. The tissues were embedded in OCT (Tissue-Tek) and sectioned into 35 μm-thick sections with a freezing microtome. Next, the tissue samples were permeabilized in 0.3% Triton, blocked in 5% donkey serum, and incubated with primary and secondary antibodies. The tissue slices were then incubated in an anti-fluorescence quenching agent.

### Image analysis

Immunostained tissues were imaged using a Zeiss LMS710 confocal microscope (Zeiss, Jena, Germany). The fluorescence was collected as Z stacks with sequential wavelength acquisition. Quantification was performed using NIH ImageJ software (National Institute of Health, Bethesda, MD, USA). To determine the colocalization of α-BTX and synaptophysin, the protein immunostaining intensity was measured. Regions of interest corresponding to pre-synaptic staining were manually selected on α-BTX images and reported on α-BTX and synaptophysin channels for intensity measurements. If the synaptophysin channels fluorescence intensity was three times higher than the background fluorescence intensity, we considered the two channels co-localized. Then, we calculated the co-localized area over the total area (α-BTX positive area).

### Antibodies

The α-BTX (tetramethylrhodamine conjugate) antibody (Invitrogen, T1175), synaptophysin antibody (Santa Cruz Biotechnology, sc-17750), Bip antibody (CST, 3177), choline acetyltransferase (Abcam, ab18736), REEP1 antibody (Sigma-Aldrich, SAB2101976), and NeuN antibody (Abcam, ab177487) were used for immunofluorescence. The REEP1 antibody (Sigma-Aldrich, SAB2101976) was also used for western blotting.

### Electron microscopy

Mice were anaesthetized and perfused with 4% PFA and 2.5% glutaraldehyde in 0.1 M sodium cacodylate buffer. Tissues were collected from both WT and REEP1^−/−^ mice and postfixed with 2% OsO_4_ for 2 h. We used 4% uranyl acetate to stain the membranes. After dehydration in a graded series of alcohols, the sciatic nerve and femoral nerve fibers were incubated in propylene oxide and Spurr's resin and then embedded in Spurr's resin. Ultrastructural images were obtained with a transmission electron microscope (TEM) (Tecnai G2 20 Twin, FEI) and analyzed by ImageJ.

### Administration of salubrinal

Salubrinal (Selleck) was reconstituted in phosphate buffered saline (PBS) containing 1% DMSO, and the solution was filtered using a 0.2 μm sterile syringe filter. Then, 0.5 mg/kg·salubrinal was administered by intraperitoneal injection once every day for 4 weeks. The dose was determined based on previous research (Rani et al., 2017).

### Statistical evaluation

Data were collected and statistically analyzed using GraphPad Prism 7.0 software. All values represent the mean±s.e.m. Statistical significance between groups was assessed by Student's *t*-test and two-way ANOVA with Sidak's multiple comparisons test. A *P*-value of less than 0.05 indicated statistical significance. The symbols used are as follows: *P<0.05, **P<0.01, and ***P<0.001.

## Supplementary Material

Supplementary information
