# Supplementary material for: Inhibition of ER stress improves progressive motor deficits in a REEP1-null mouse model of hereditary spastic paraplegia
Source: Biol Open. 2020 Sep 29;9(9):bio054296. doi: 10.1242/bio.054296 (PMC7541344; doi:10.1242/bio.054296)
Supplement: Supplementary information [file biolopen-9-054296-s1.pdf]

supplementary materials

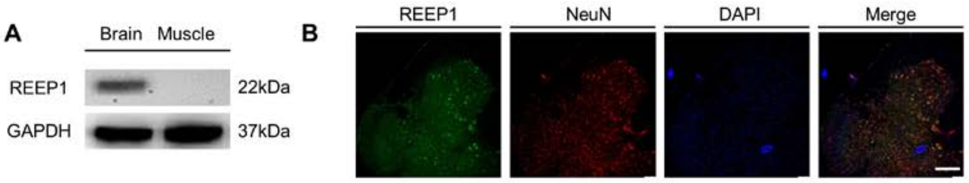

Figure S1.

(A) REEP1 expressed in the brain, but not in the muscle. (B) Co-stained REEP1, NeuN and DAPI to verify REEP1 expression in the lower motor neurons. REEP1 (green), NeuN (red), DAPI (blue). Scale bars: 50 µm

Biology Open • Supplementary information
